# Effect of High-Fat Diet  on  the Intestinal Flora in Letrozole-Induced Polycystic Ovary Syndrome Rats

**DOI:** 10.1155/2021/6674965

**Published:** 2021-06-25

**Authors:** Yan-Hua Zheng, Ying Xu, Hong-Xia Ma, Cheng-Jie Liang, Tong Yang

**Affiliations:** ^1^Department of Traditional Chinese Medicine, The Second Affiliated Hospital of Guangzhou Medical University, Guangzhou 510260, Guangdong, China; ^2^Department of Nutrition, Fujian Medical University Union Hospital, Fuzhou 350001, Fujian, China; ^3^Department of Traditional Chinese Medicine, The First Affiliated Hospital of Guangzhou Medical University, Guangzhou 510120, Guangdong, China; ^4^Animal Experiment Center, Guangzhou Medical University, Guangzhou 511436, Guangdong, China; ^5^Department of Pathology, The Second Affiliated Hospital of Guangzhou Medical University, Guangzhou 510260, Guangdong, China

## Abstract

**Aim:**

The aim of this study was to explore whether letrozole and high-fat diets (HFD) can induce obese insulin-resistant polycystic ovary syndrome (PCOS) with intestinal flora dysbiosis in a rat model. We compared the changes in the intestinal flora of letrozole-induced rats fed with HFD or normal chow, to explore the effects of HFD and letrozole independently and synergistically on the intestinal flora.

**Methods:**

Five-week-old female Sprague Dawley (SD) rats were divided into four groups: control (C) group fed with regular diet; L1 group administered with letrozole and fed with regular diet; L2 group received letrozole and fed with HFD; and HFD group fed with HFD. At the end of the experiment, ovarian morphology, hormones, metabolism, oxidative stress, and inflammatory status of all rats were studied. 16S rDNA high-throughput sequencing was used to profile microbial communities, and various multivariate analysis approaches were used to quantitate microbial composition, abundance, and diversity.

**Results:**

Compared to the C group, the increased plasma fasting insulin and glucose, HOMA-IR, triglyceride, testosterone, and malondialdehyde were significantly higher in the L2 group, while high-density lipoprotein cholesterol was significantly lower in the L1 group and L2 group. The indices of Chao1 and the Abundance-based Coverage Estimator (ACE) (*α*-diversity) in the L2 and HFD groups were significantly lower than that in the C group. Bray–Curtis dissimilarity based principal coordinate analysis (PCoA) plots and analysis of similarities (ANOSIM) test showed obvious separations between the L2 group and C group, between the HFD group and C group, and between the L2 and HFD groups. At the phylum level, *Firmicutes* and ratio of *Firmicutes* and *Bacteroidetes* (F/B ratio) were increased in the L2 group; *Bacteroidetes* was decreased in the L2 and HFD groups. No significant differences in bacterial abundance between the C group and L1 group were observed at the phylum level. Based on linear discriminant analysis (LDA) effect size (LEfSe) analysis, the bacterial genera (the relative abundance > 0.1%, LDA > 3, *p* < 0.05) were selected as candidate bacterial signatures. They showed that the abundance of *Vibrio* was significantly increased in the L1 group; *Bacteroides* and *Phascolarctobacterium* were enriched in the HFD group, and *Bacteroides*, *Phascolarctobacterium*, *Blautia*, *Parabacteroides*, *Akkermansia [Ruminococcus]_torques_group*, and *Anaerotruncus* were enriched in the L2 group.

**Conclusion:**

The effect of letrozole on intestinal flora was not significant as HFD. HFD could destroy the balance of intestinal flora and aggravate the intestinal flora dysbiosis in PCOS. Letrozole-induced rats fed with HFD have many characteristics like human PCOS, including some metabolic disorders and intestinal flora dysbiosis. The dysbiosis was characterized by an increased Firmicutes/Bacteroidetes ratio, an expansion of Firmicutes, a contraction of Bacteroidetes, and the decreased microbial richness. Beta-diversity also showed significant differences in intestinal microflora, compared with control rats.

## 1. Background

PCOS is a common endocrine and metabolic syndrome among women of reproductive age [1]. Alterations in intestinal flora composition or “dysbiosis” have been implicated in the pathological development of PCOS [2]. Testosterone (T) concentration may affect the composition of the intestinal microbial community, and several studies have found that changes in the intestinal microbial community in PCOS women are related to hyperandrogenism and low *α*-diversity compared with the control group [3, 4]. Intestinal flora may play a pathogenic role in regulating energy balance and participate in the development and process of obesity and metabolic diseases [5]. Intestinal flora dysbiosis can interfere with normal follicular development by triggering a chronic inflammatory reaction and insulin resistance (IR), which is closely linked to the occurrence and development of PCOS [6]. The composition of the intestinal microflora is affected by many environmental factors. Diet is considered to be one of the most important environmental factors affecting the composition of the intestinal microbial community [7]. Diet-induced obesity is related to a variety of metabolic and reproductive disorders, including PCOS [8]. The heterogeneity of PCOS is frequently reflected in many animal models. Therefore, if a rat model can show not only the characteristics of ovarian and metabolic syndrome but also the imbalance of intestinal flora, it would be valuable for further study of new PCOS therapy. Letrozole is a nonsteroidal aromatase inhibitor, which can increase testosterone levels and reduce estrogen levels by inhibiting the conversion of testosterone to estrogen [9]. According to the report, the letrozole-induced model recapitulates many histological and biochemical aspects consistent with human PCOS [10]. In the present study, female Sprague Dawley (SD) rats were given oral letrozole to establish a model of PCOS and fed with a regular diet or HFD. We studied the reproduction, metabolism, and intestinal flora community of these rats. The findings of this study may also help us better understand the effects of HFD and letrozole on the intestinal flora of PCOS.

## 2. Materials and Methods

### 2.1. Animals

At the beginning of the experiment, twenty-one female specific pathogen-free (SPF) SD rats aged 5 weeks came from the Experimental Animal Science Department of Guangzhou University of Chinese Medicine, Guangzhou, China (License number SCXK-2016-0168). This experiment was approved by the Institutional Animal Care and Use Committee of Guangzhou Medical University and was conducted in strict accordance with the guidelines for Ethical Review of the Welfare of Experimental Animals (GB/T 35892-2018) in China. All rats were provided with humane care in a temperature-controlled room with a 12 hr light/dark cycle (lights on 07:00–19:00) and ad libitum access to food and water in their cages (22°C–24°C and 60% humidity).

### 2.2. Study Procedure

Rats were adaptively fed for one week and then divided into four groups. The control group (*n* = 5) received an aqueous solution of 1% carboxymethyl cellulose sodium (CMC) and consumed with normal chow (Research Diets GB 14924.3-2010, energy%: 67% carbohydrate, 21% protein, 12% fat, and total 3.45 kcal/g, provided by Guangdong Medical Laboratory Animal Center). The PCOS rat model in our study was established according to the method of Kafali Het al. [10]. PCOS 1 group (L1, *n* = 5) was fed with regular diet and administered with letrozole (Target Mol, American, 1 mg/kg) dissolved in solution CMC1% [10]; PCOS 2 group (L2, *n* = 6) was fed with HFD (D12492, energy%: 60% fat, 20% carbohydrates, and 20% protein, 5.24 kcal/g, provided by Guangdong Medical Laboratory Animal Center) and administered with letrozole (1 mg/kg) dissolved in solution CMC 1%; HFD group (*n* = 5) received an aqueous solution of CMC 1% and consumed with HFD. All doses were given orally via gavage, for 8 consecutive weeks, and vaginal cytology analysis was done until the end.

### 2.3. Vaginal Smear

The stage of the estrus cycle was determined by the main cell type in vaginal smears, which started from 6 weeks of age to the end of the experiment every day [11]. All rats were collected daily by using a dropper filled with normal saline (0.9% NaCl).

### 2.4. Measurement of Hormone Profile and Biochemical Indexes

The rats were anesthetized with 2% pentobarbital sodium (100 *μ*g/g of body weight). After the ovaries were taken out, the chest was opened; about 4 ml of blood was taken from the heart. The rats were sacrificed by overdose pentobarbital sodium. Blood was withdrawn through orbital sinus in a tube and separated by 10 min centrifugation (3,000 revolutions/min) at 4°C. Supernatant containing serum was separated and stored immediately at −20°C until analyzed for biochemical and hormonal analysis. Fasting blood glucose (FBG) was analyzed by GOD-PAP. Testosterone (T), superoxide dismutase (SOD), malondialdehyde (MDA), interleukin-22(IL-22), fasting insulin (FINS), luteinizing hormone (LH), follicle-stimulating hormone (FSH), lipopolysaccharide (LPS), Toll-like receptor 4 (TLR4), and tumor necrosis factor-*α* (TNF-*α*) were determined using enzyme-linked immunosorbent assay (ELISA) kit (Mlbio, Shanghai, China). Low-density lipoprotein (LDL) cholesterol, high-density lipoprotein (HDL) cholesterol, total cholesterol (TC), and triglyceride (TG) levels were measured using Chemistry Analyzer (UniCelDxC 600 Synchron, Beckman Coulter, USA). IR was appraised with the homeostasis model assessment of insulin resistance (HOMA-IR) method. HOMA-IR was calculated using the following formula: HOMA-IR = FBG (mmol/L) *∗*FINS (mU/L)/22.5 [12].

### 2.5. Sample Collection

Fresh stool samples were extracted from the colons of all rats, collected into 1.5 ml sterile EP tubes, then frozen in liquid nitrogen quickly, and stored at −80°C until further analysis. The right ovary of the rat was fixed in 4% paraformaldehyde and embedded in paraffin. 5 *μ*m thick sections were prepared and stained with hematoxylin-eosin (HE) and histoanatomical changes were observed and photographed under a light microscope (BX-51, Olympus, Tokyo, Japan, at X40 magnification).

### 2.6. 16S rDNA Sequencing Data Analysis

The fecal microbiome for 21 fecal samples was collected from the rats in the four groups. The 16S rDNA high-throughput sequencing (V3-V4 region) was performed using an Illumina MiSeq platform. After assembly, quality filtering, and the random extraction of sequences at 97% similarity, the operational taxonomic units (OTUs) for species classification were obtained. The Chao1, ACE, Simpson, and Shannon indexes were calculated to analyze *α*-diversity. We used Bray–Curtis dissimilarity to analyze and compare the similarity of the gut microbial communities (*β*-diversity). Analysis of similarities (ANOSIM) test was used to check whether the differences between groups were significantly greater than those within groups. A principal coordinate analysis (PCoA) plot was used to visualize whether the groups have significantly different microbial communities. Linear discriminant analysis effect size (LEfSe) analysis coupled with the Kruskal–Wallis rank-sum test was performed to identify the microbial differences among all groups. Note that while a log-transformed LDA score of 2 was used as a threshold for identification of significant taxa, the LDA >3.0 was set as the threshold for selection of features.

### 2.7. Statistical Analyses

Most statistical evaluations were performed with SPSS 21.0 for Windows (SPSS Inc., Chicago, IL, United States). All data were presented as mean ± SEM. One-way ANOVA was used to determine the significance, and *p* < 0.05 was considered significant. When the ANOVA revealed significant differences among four groups, a post hoc analysis was performed by a Tukey honest significant difference test. The Kruskal–Wallis test was used for not normally distributed values. *α*-diversity was analyzed using Chao1, ACE, Shannon, and Simpson diversity indices. These indexes were calculated for the samples using QIIME (v1.7.0) based on the rarefied OTU counts and were displayed using R software (v2.15.3). *β*-Diversity analysis was used to evaluate differences in the species complexity between samples, and beta-diversity based on Bray–Curtis dissimilarity was calculated using QIIME software (v1.7.0) based on the rarefied OTU counts. The microbiota features differentiating the fecal microbiota were characterized using the LEfSe method for biomarker discovery, which uses the Kruskal–Wallis rank-sum test to detect features with significantly different abundance levels between assigned taxa and performs an LDA to estimate the effect size of each feature.

## 3. Results

### 3.1. Reproductive and Metabolic Parameters

**Figure 1:**
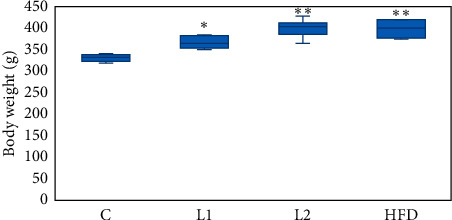
The body weights were measured at the end of experiment.  ^*∗*^Compared with the C group, *p* < 0.05;  ^*∗∗*^compared with the C group, *p* < 0.01.

**Figure 2:**
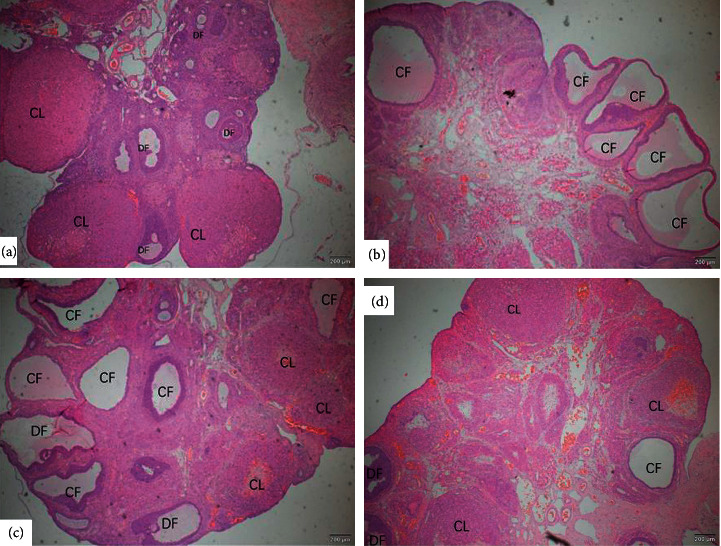
Photomicrographs of representative ovarian cross section from four groups: (a) C group, (b) L1 group, (c) L2 group, and (d) HFD group. DF: developing follicles; CL: corpus luteum; CF: cystic follicles.

Body weight was measured weekly. The weight of rats in the L1, L2, and HFD groups increased more than that in the C group (*p* < 0.01) (Figure 1). As seen in Table 1, compared with the C group, the increased plasma fasting insulin and glucose, HOMA-IR, TG, T, and MDA  were significantly higher in the L2 group (*p* < 0.01 or *p* < 0.05), while HDL-C was lower in the L1 group and L2 group (*p* < 0.05). The level of LPS was significantly higher in the HFD group than in the C group (*p* < 0.05). The reproductive function of the ovaries was evaluated based on estrous cyclicity, follicle number, and follicle morphology. Rats in the C and HFD groups showed regular cycles of 4-5 days complete with the proestrus, estrus, metestrus, and diestrus stages. Ovaries from the C and HFD groups exhibited follicles in various stages of development, including some fresh corpora lutea. At the end of the study, rats in the L1 and L2 groups had irregular cycles and were in the diestrus stage which mainly showed leukocytes. Hematoxylin-eosin (HE) staining was conducted to evaluate the ovary structure. HE staining indicated that the ovaries of the L1 and L2 group had a high incidence of subcapsular ovarian cyst together with incomplete luteinization and decreased number of corpora lutea (Figures 2(a)–2(d)).

### 3.2. Diversity of the Intestinal Flora

**Figure 3:**
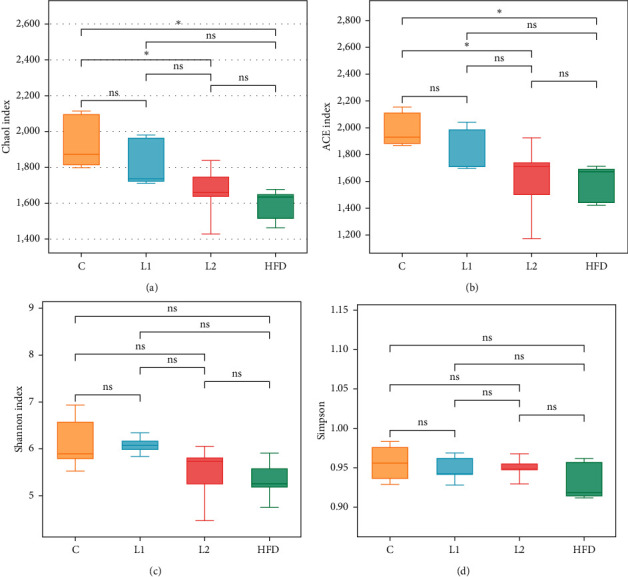
Alpha-diversity analysis of the species distribution. (a) Chao1 index. (b) ACE index. (c) Shannon value. (d) Simpson value.  ^*∗*^*p* < 0.05, ns: not significant, *p* < 0.05.

**Figure 4:**
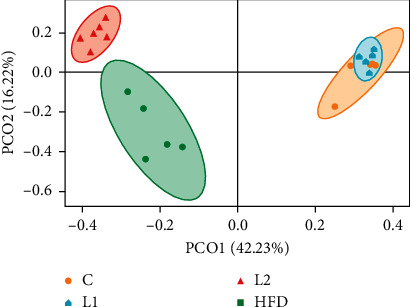
Principal coordinate analysis (PCoA) plot of bacterial community composition at the OTU level to evaluate the similarities among the groups. Each dot represents the bacterial community of the sample.

**Figure 5:**
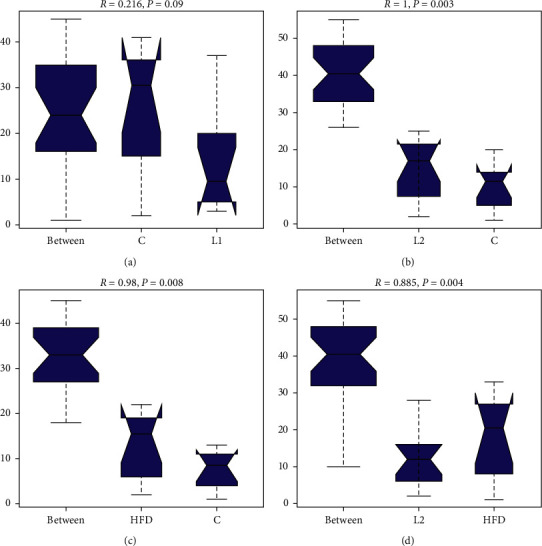
Analysis of similarities (ANOSIM) plot showing dissimilarity between groups. (a) Between C and L1 groups. (b) Between L2 and C groups. (c) Between HFD and C groups. (d) Between L2 and HFD groups. *p* value is a measure of the significance of the trend between groups. *R*-value is a measure of the strength of the factors on the samples. *R*-value close to 1 indicates a high separation between groups.

OTU-level alpha-diversity metrics (ACE and Chao1) revealed significantly lower diversity and richness in the L2 and HFD groups. Compared with the C group, the ACE and Chao1 indices in the L2 and HFD groups were significantly decreased (*p* < 0.05) (Figures 3(a)–3(d)). However, there were no significant differences in the Shannon or Simpson index between the groups. PCoA plot revealed distinct clustering of C group that separated from both the L2 and HFD groups (Figure 4). The significance of differences was confirmed by the ANOSIM, C and L2 groups (*R* = 1, *p*=0.003) (Figure 5(b)), C and HFD group (*R* = 0.98, *p*=0.008) (Figure 5(c)), and L2 and HFD groups (*R* = 0.885, *p*=0.004) (Figure 5(d)), and *R* > 0.5 implies that separation between groups is good and intergroup variation is significantly greater than intragroup variations.

### 3.3. The Composition of Intestinal Flora

**Figure 6:**
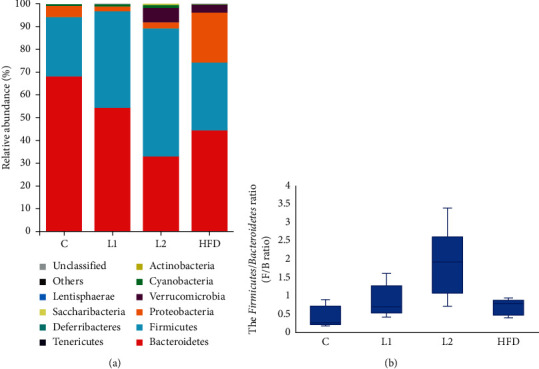
Comparison of microbiota composition at the phylum level. (a) A bar plot about relative abundance (%) of bacterial taxa. (b) The relative abundance of *Bacteroidetes* and *Firmicutes*, and the *Firmicutes*/*Bacteroidetes* ratio (F/B ratio).  ^*∗∗*^Compared with the C group, *p* < 0.01.

**Figure 7:**
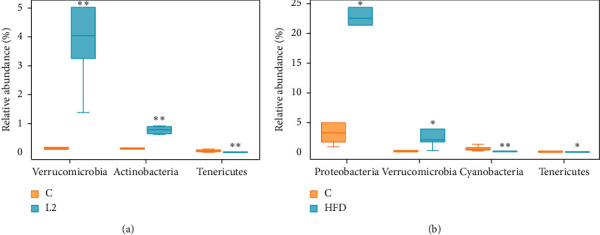
Boxplot of comparing the relative abundance between groups at the phylum level. (a) Between C and L2 groups. (b) Between C and HFD groups.  ^*∗*^Compared with the C group, *p* < 0.05;  ^*∗∗*^compared with the C group, *p* < 0.01.

We evaluate the intestinal flora composition by comparing the relative abundances at the phylum and genera levels. The 10 major bacterial clades from the gut bacterial profiles of the groups at phylum level are represented in Figure 6(a). The phyla *Firmicutes*, *Bacteroidetes*, *Proteobacteria*, *Verrucomicrobia*, and *Actinobacteria* dominate the intestinal flora community. Compared with the C group, the relative abundance of *Firmicutes* and ratio of *Firmicutes* and *Bacteroidetes* (F/B ratio) were increased, and *Bacteroidetes* was decreased in the L2 group (*p* < 0.01). And the relative abundance of *Bacteroidetes* was also decreased significantly in the HFD group (Figure 6(b)). Moreover (Figure 7(a) and 7(b)), compared with the C group, the relative abundance of *Verrucomicrobia* and *Actinobacteria* was enriched, and *Tenericutes* was decreased in the L2 group (*p* < 0.05). The relative abundance of *Proteobacteria* and *Verrucomicrobia* (*p* < 0.05) was increased in the HFD group, while *Tenericutes* and *Cyanobacteria* (*p* < 0.01) were decreased, compared with the C group. No significant differences between the C group and L1 group were observed at phylum level.

**Figure 8:**
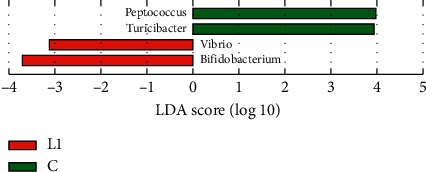
LDA along with effect size measurements was applied to present the enriched bacterial genera in the L1 group (red) and C group (green).

**Figure 9:**
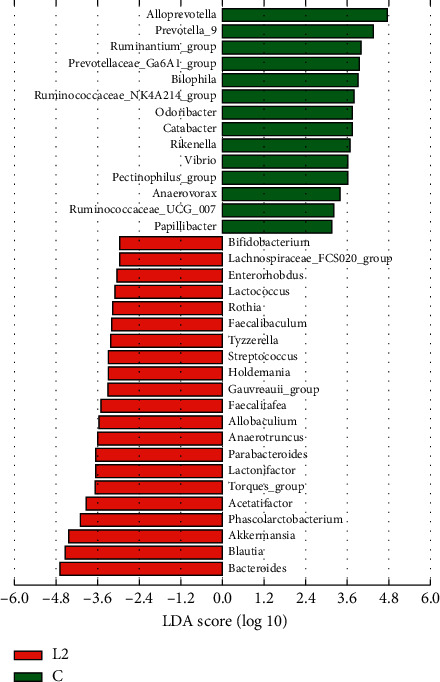
LDA along with effect size measurements was applied to present the enriched bacterial genera in the L2 group (red) and C group (green).

**Figure 10:**
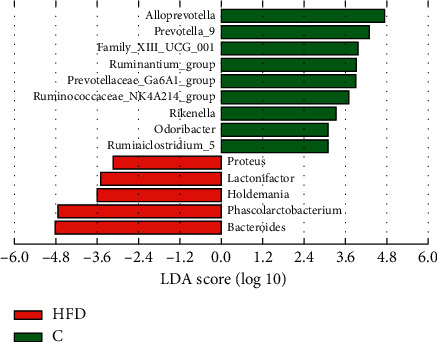
LDA along with effect size measurements was applied to present the enriched bacterial genera in the HFD group (red) and C group (green).

**Figure 11:**
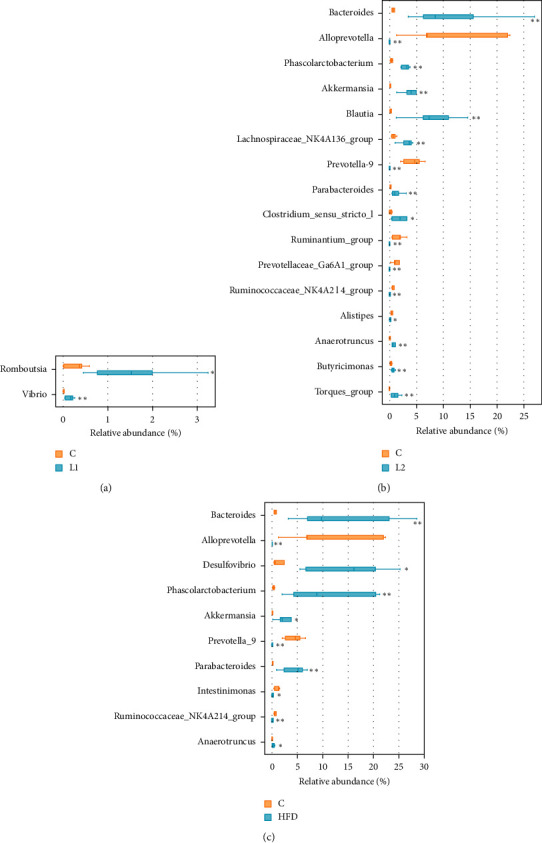
Comparisons of the relative abundances (%) of bacterial genera between groups. (a) Between C and L1 group. (b) Between C and L2. (c) Between C and HFD group.  ^*∗*^Compared with the C group, *p* < 0.05;  ^*∗∗*^compared with the C group, *p* < 0.01.

In this work, we used the LEfSe method to identify significant, differentially abundant microbiome. At the genus level, the results showed that four genera were distinctively represented between the L1 group and C group, with two (*Peptococcus* and *Turicibacter*) being abundant in the C group, and two (*Vibrio* and *Bifidobacterium*) being abundant in the L1 group (Figure 8). As seen in Figure 9, thirty-five genera were obviously representative between the L2 group and C group, with fourteen (*Alloprevotella*, *Prevotella_*9, *ruminantium_group, Bilophila*, *Prevotellaceae_Ga*6*A*1*_group*, *Ruminococcaceae_NK*4*A*214*_group*, *Odoribacter*, *Catabacter*, *Rikenella*, *Vibrio*, *pectinophllus_group*, *Anaerovorax*, *Ruminococcaceae_UCG-*007, and *Papillibacter*) being abundant in the C group, and twenty-one (*Bacteroides*, *Blautia*, *Akkermansia*, *Phascolarctobacterium*, *Parabacteroides*, *[Ruminococcus]_torques_group*, *Anaerotruncus*, *Allobaculum*, *Faecalitalea*, *Streptococcus*, *Tyzzerella*, *Faecalibaculum*, *Enterorhabdus*, *Bifidobacterium*, *Rothia*, *Lactococcus*, *Lachnospiraceae_FCS*020*_group*, *Holdemania, gauvreauii_group*, *Lactonifactor*, and *Acetatifactor*) being abundant in L2 group. Fourteen genera differed dramatically between the HFD group and C group, the proportions of the *Alloprevotella*, *Prevotella_9*, *Family_XIII_UCG_group*, *ruminantium_group*, *Prevotellaceae_Ga*6*A*1*_group*, *Ruminococcaceae_NK4A214_group*, *Rikenella*, *Odoribacter*, and *Ruminiclostridium_*5 genera were decreased, whereas the proportions of the *Proteus*, *Lactonifactor*, *Holdemania*, *Phascolarctobacterium*, and *Bacteroides* were increased in the HFD group samples (Figure 10). Subsequently, the genera above with the average relative abundance >0.1% were analyzed by the Wilcoxon rank-sum test between the C, L1, L2, and HFD groups (Figures 11(a)–11(c)). Compared with the C group, the relative abundance of Romboutsia and Vibrio was increased in the L1 group; *Bacteroides*, *Blautia*, *Akkermansia*, *Phascolarctobacterium*, *Parabacteroides*, *Clostridium_sensu_sticto_1*, *[Ruminococcus]_torques_group*, *Anaerotruncus*, and *Butyricimonas* were enriched in the L2 group significantly, while the proportions of the *Alloprevotella, Prevotella_9, ruminantium_group, Prevotellaceae_Ga6A1_group, Ruminococcaceae_NK4A214_group*, and *Alistipes* were decreased. *Bacteroides*, *Desulfovibrio*, *Phascolarctobacterium*, *Akkermansia*, *Parabacteroides*, and *Anaerotruncus* were increased in the HFD group; *Alloprevotella*, *Prevotella_9*, *Intestinimonas*, and *Ruminococcaceae_NK4A214_group* were decreased.

## 4. Discussion

PCOS is the most common endocrine disorder, with many complications such as obesity and IR. The rats in the L1 and L2 groups gained more weight than the controls and showed some reproductive phenotypes of PCOS, including hyperandrogenism, anovulation (indicated by a lack of corpora lutea in the ovaries), and the appearance of cystic ovarian follicles. Combined with HFD, the metabolic disorder seemed to aggravate, the fasting insulin and glucose, HOMA-IR, and TG were significantly elevated, and HDL-C was reduced in the L2 group, compared with the C group. And the concentration of MDA was also raised in the L2 group. It is known that excessive intake of fat may affect the intestinal flora, increase circulating LPS, trigger downstream inflammatory events, and increase the risk of long-term low-level systemic inflammation, obesity, and IR [13–18]. Diet is considered to be one of the most critical environmental factors for shaping intestinal flora structures. HFD can influence the intestinal flora directly, and increase the circulatory LPS [19]. In our study, the rats in the L2 and HFD groups were fed with HFD, and the concentration of LPS in the HFD group increased significantly, but there was no such significant change in the L2 group. HFD feeding seemed to interfere with the *α*- and *β*-diversity of the microbial community more significantly than letrozole. OTU-level *α*-diversity metrics (ACE and Chao1) revealed significantly lower richness in the L2 and HFD groups. It has been proved that individuals with low microbial richness are more prone to obesity, IR, and dyslipidemia [20]. After correcting for age and sex, OTU richness was negatively correlated with BMI and TG, but positively correlated with HDL-C [21]. In line with the fact that HDL-C was decreased in the L2 and HFD groups, the fasting insulin and blood glucose, TG, and HOMA-IR were elevated significantly in the L2 group. Significant differences were found in *β*-diversity between the L2 group and C group, between the HFD group and C group, and L2 and HFD groups, but they were not found between the C group and L1 group. The above results suggested that the microbiota community in the HFD and L2 groups were significantly different compared to the C group. And the microbial environment was not changed significantly after treating with letrozole alone but changed obviously after feeding with HFD.

All predominant phyla, including *Firmicutes*, *Bacteroidetes*, *Proteobacteria*, *Verrucomicrobia*, and *Actinobacteria*, were largely consistent in different groups, and different relative abundances could be observed. A decrease of *Bacteroidetes* along with an increase of *Firmicutes* resulted in an increased F/B ratio in the L2 group. An increased F/B ratio has been widely considered a signature of gut dysbiosis [22]. Gut microbial dysbiosis has been associated with inflammatory and metabolic disorders [23] and obesity [24]. The results showed that the rats in the L2 group had higher body weight, fasting insulin, fasting blood glucose, and HOMA-IR than in the C group. It was reported that *Bacteroidetes*-rich communities have a protective effect on blood glucose level [25] and play a protective role in intestinal inflammation [26]. A reduction of *Bacteroides* is related to some metabolic diseases, such as diabetes and cardiac disease [27]. Increased *Firmicutes* was correlated with obesity [28]. The relative abundance of *Actinobacteria* was also enriched in the L2 group. The function of *Actinobacteria* in gut microbiota was not thoroughly understood. It was reported that *Actinobacteria* was increased in human adults with type II diabetes [29]. In a survey about thin and obese twins, a higher level of *Actinobacteria* in the gut was found in obese subjects [30]. Intriguingly, different from our study, Lindheim et al. found a reduced relative abundance of bacteria from the *Actinobacteria* phylum in PCOS patients [31]. The relative abundance of *Verrucomicrobia* was enriched, and *Tenericutes* was decreased in the L2 group and HFD group. *Tenericutes* phylum was found enriched in healthy individuals compared with metabolic syndrome patients [32]. In Europeans, PCOS was reported to be related to the decrease of relative abundance of *Tenericutes* [33]. And the decrease abundance of *Tenericutes* was observed in intestinal dysbiosis of rats due to inflammatory conditions [34], as well as the increase of *Verrucomicrobia* [35]. *Proteobacteria* phylum, which includes a wide variety of pathogens, was also more abundant in the HFD group. The phylum *Proteobacteria* is the most unstable over time among the main phyla in the intestinal flora [36]. The increased prevalence of *Proteobacteria* reflects dysbiosis or an unstable intestinal flora community structure [37]. Therefore, intake of HFD could increase the relative abundance of *Proteobacteria* and interfere with the stability of the microbial community. Letrozole alone may not significantly affect intestinal stability as HFD, but HFD combined with letrozole have synergistic effects on altering the composition and structure of intestinal flora.

At the genus level, we used the LEfSe method to compare the intestinal flora compositions of the control group to the other three groups and identify the specific bacterial taxa. The larger the LDA score, the more significant the difference between groups. Based on the LDA and Wilcoxon rank-sum test, the bacterial genera (the relative abundance > 0.1%, LDA > 3, *p* < 0.05) were selected as candidate bacterial signatures. *Vibrio* was enriched in the L1 group as a biomarker. *Vibrio* is known as an opportunistic bacterial pathogen which might increase host susceptibility [38]. The HFD group was characterized by a higher content of *Bacteroides* and *Phascolarctobacterium*. Intestinal microbial communities are known to be affected by diet. Dietary habits such as foods with saturated fats and animal protein can lead to a high prevalence of *Bacteroides* [39, 40]. A higher abundance of *Bacteroides* was observed in Japanese participants who consumed a diet of animal origin in comparison to Indian adults who consumed a more plant-based diet [41]. *Bacteroides* is also one of the major lineages of bacteria and associated with gut inflammation [42, 43]. *Bacteroides* species are most commonly found in mixed infections [44]. Moreover, increased levels of *Bacteroides* were negatively correlated with energy intake and adiposity [45]. *Phascolarctobacterium* can produce short-chain, which is positively correlated with the metabolic status in the host [46, 47]. It was also negatively correlated with many pathways, including environmental information processing and metabolism [48]. *Phascolarctobacterium* is related to both insulin sensitivity and secretion [49], and a higher abundance of *Phascolarctobacterium* was observed in women with metabolic syndrome [50].

Letrozole may have enhanced the effects of HFD on intestinal flora imbalance. In addition to enrichment of *Bacteroides* and *Phascolarctobacterium*, the relative abundance of *Blautia*, *Parabacteroides*, *[Ruminococcus]_torques_group*, *Akkermansia*, and *Anaerotruncus* was presented abundant in the L1 group. *Blautia*, which is considered to be essential for a healthy status [51], may contribute to the alleviation of inflammation, IR, and obesity by reducing the intestinal endotoxins into the blood [52]. However, *Blautia* has been found increased in disease groups in three out of four cross-sectional studies for type 2 diabetes [53]. As a producer of acetate, *Blautia* can drive the release of insulin and promote metabolic syndromes, such as hypertriglyceridemia, fatty liver, and IR [54]. The relative abundance level of *Blautia* was positively correlated with bowel symptoms and increased in patients with irritable bowel syndrome [55]. *Blautia* has been shown to be associated with metabolites reflecting an unhealthy metabolic state in individuals with a high BMI [56]. Many studies illustrated that *Blautia* can drive insulin release and promote metabolic syndromes, such as hypertriglyceridemia, fatty liver disease, and IR [53, 57]. Patients with type 2 diabetes and glucose intolerance had greater numbers of *Blautia* [58]. *Blautia* was also positively correlated with indicators of bodyweight (including waistline and body mass index) and serum lipids (including LDL-C, TC, and TG) [59]. *Parabacteroides* enrichment may alter gene expression in pathways associated with metabolic function, neurodegenerative disease, and dopaminergic signaling [60]. Some studies have reported that *Parabacteroides* is negatively correlated with metabolic disorders [61, 62]. *[Ruminococcus]_torques_group* may alter fat metabolism; low abundance of *Ruminococcus_torques_group* is beneficial for the control of body fat and promotes the effects of resistant starch on abdominal adiposity [63]. *Ruminococcus]_torques_group* was also reported to be associated with inflammatory bowel disease [64], and more abundant in subjects consuming the proinflammatory diets [65]. *Anaerotruncus* is a conditional pathogenic bacterium and reported to be linked to hepatic cirrhosis with Holdemania and Dorea and type 1 diabetes, but not specific to IR [66]. In addition, in the mouse study, the relative abundance of *Anaerotruncus* species is also related to aging, age-related inflammation, and the increase of proinflammatory chemokines [67]. It is reported that *Akkermansia* has both regulatory and inflammatory properties [68]. The enrichment of *Akkermansia* has been found to be inversely associated with obesity and diabetes mellitus [69]. *Akkermansia* has previously been reported to associate with improved metabolic health, and the introduction of the *Akkermansia* into the gut of diet-induced obese mice may improve the host glucose homeostasis [70].

The results indicated that letrozole combined with HFD apparently changed microbial diversity and composition, which can influence the host metabolism mainly through various mechanisms, including getting more energy from the diet, disturbing metabolism, and immunologic function.

## 5. Conclusion

Letrozole has synergistic effects with HFD on intestinal flora dysbiosis. The consumption of HFD might contribute to accelerating the progression of oxidative stress status, aggravating metabolic disorder in PCOS. The present findings support the notion that the letrozole- and HFD-induced rat model has many characteristics of human PCOS, including some metabolic disorders and intestinal flora dysbiosis. The rat model of PCOS may provide a useful tool for evaluating the efficacy and mechanism of new monotherapy or drug combinations in treating PCOS.

## 6. Limitation

Because the biological samples of the microbial community obtained in this study were limited, the effects of letrozole on the intestinal microbial community may not be significant. The search for intestinal microflora via stool carries specific limitations. Stool may represent lower intestinal microflora, but composition differs between upper and lower intestine [72].

## Figures and Tables

**Table 1:** Comparison of biochemical parameters among groups.

	C	L1	L2	HFD	ANOVA	Tukey HSD (adjusted for multiple comparisons)		
	*N* = 5	*N* = 6	*N* = 6	*N* = 5	*p* Value	P2	P3	P4
LH (mIU/ml)	5.30 ± 0.81	5.51 ± 0.78	5.38 ± 0.65	6.06 ± 0.73	0.372	0.966	0.894	0.331
FSH (mIU/ml)	7.36 ± 1.13	7.00 ± 0.98	6.91 ± 0.72	7.71 ± 1.18	0.558	0.945	0.876	0.942
T (pg/mL)	26.46 ± 3.04	38.92 ± 9.35	39.50 ± 7.08	33.72 ± 9.35	0.23	0.05	0.022	0.344
FINS (mU/L)	2.49 ± 0.46	2.17 ± 0.40	3.73 ± 0.64	2.61 ± 0.35	0	0.742	0.002	0.982
FBG (mmol/L)	5.46 ± 0.83	5.5 ± 0.1.37	8.33 ± 1.03	6.84 ± 1.75	0.003	1	0.006	0.329
HOMA-IR	0.59 ± 0.07	0.54 ± 0.19	1.34 ± 0.42	0.79 ± 0.25	0	0.991	0.001	0.655
HDL-C (mmol/L)	1.15 ± 0.22	0.85 ± 0.18	0.83 ± 0.11	0.97 ± 0.09	0.02	0.044	0.02	0.302
LDL-C (mmol/L)	0.41 ± 0.13	0.43 ± 0.10	0.42 ± 0.02	0.38 ± 0.04	0.843	0.992	0.997	0.952
TG (mmol/L)	0.46 ± 0.08	0.46 ± 0.10	0.72 ± 0.15	0.53 ± 0.12	0.004	1	0.008	0.799
TC (mmol/L)	1.25 ± 0.41	1.44 ± 0.38	1.64 ± 0.20	1.44 ± 0.19	0.247	0.771	0.187	0.753
TLR4 (ng/mL)	3.23 ± 0.40	3.35 ± 0.43	3.48 ± 0.36	3.61 ± 0.53	0.611	0.971	0.778	0.526
LPS (EU/L)	93.00 ± 8.57	105.9 ± 14.7	115.6 ± 32.6	137.36 ± 30.26	0.049	0.842	0.418	0.039
SCAF (pg/ml)	29.47 ± 2.16	34.51 ± 16.6	30.90 ± 6.56	27.59 ± 1.32	0.653	0.816	0.993	0.986
SOD (U/ml)	24.96 ± 2.95	18.93 ± 10.1	19.67 ± 4.08	23.35 ± 4.33	0.342	0.425	0.463	0.972
MDA (nmol/ml)	0.26 ± 0.06	0.44 ± 0.13	0.46 ± 0.09	0.19 ± 0.02	0	0.071	0.022	0.32
IL-22 (pg/ml)	3.81 ± 0.57	4.09 ± 0.29	4.21 ± 0.49	3.52 ± 0.50	0.109	0.794	0.502	0.754
TNF-*α* (pg/ml)	50.1 ± 5.39	48.3 ± 7.21	54.9 ± 9.80	53.15 ± 5.31	0.427	0.98	0.684	0.91

LH: luteinizing hormone; FSH: follicle-stimulating hormone; T: testosterone; FT: free testosterone; INS: fasting insulin; FBG: fasting blood glucose; HOMA-IR: homeostasis model of assessment for insulin resistance index; HDL-C: high-density lipoprotein cholesterol; LDL-C: low-density lipoprotein cholesterol; TG: total triglyceride; TC: total cholesterol; TLR4: Toll-like receptor 4; LPS: lipopolysaccharide; SOD: superoxide dismutase; MDA: malondialdehyde; IL-22: interleukin-22; TNF-*α*: tumor necrosis factor-*α*. Data are presented as mean ± standard deviation, analyzed by one-way analysis of variance followed by the Tukey HSD test. P2: C group versus L1 group; P3: C group versus L2 group; P4: C group versus HFD group.

## Data Availability

The datasets used and/or analyzed during the current study are available from the corresponding author on reasonable request.

## Abbreviations

PCOS: Polycystic ovary syndrome
SD: Sprague Dawley
HFD: High-fat diet
PCoA: Principal coordinate analysis
IR: Insulin resistance
SPF: Specific pathogen-free
OTUs: Operational taxonomic units
LH: Luteinizing hormone
FSH: Follicle-stimulating hormone
T: Testosterone
INS: Fasting insulin
FBG: Fasting blood glucose
HOMA-IR: Homeostasis model of assessment for insulin resistance index
HDL-C: High-density lipoprotein cholesterol
LDL-C: Low-density lipoprotein cholesterol
TG: Total triglyceride
TC: Total cholesterol
TLR4: Toll-like receptor 4
LPS: Lipopolysaccharide
SCAF: Short-chain fatty acid
SOD: Superoxide dismutase
MDA: Malondialdehyde
IL-22: Interleukin-22
TNF-*α*: Tumor necrosis factor-*α*.
